# The Cadherin Superfamily in *Anopheles gambiae*: a Comparative Study With *Drosophila melanogaster*

**DOI:** 10.1002/cfg.473

**Published:** 2005-06

**Authors:** Catarina Moita, Sérgio Simões, Luís F. Moita, António Jacinto, Pedro Fernandes

**Affiliations:** 1 Instituto Gulbenkian de Ciência Rua da Quinta Grande 6 Apartado 14 Oeiras 2781-901 Portugal; 2 Center for Immunology and Inflammatory Diseases Division of Rheumatology Allergy and Immunology Massachusetts General Hospital, 149 13th Street, Room 8301 Charlestown MA 02129 USA; 3 Institute of Molecular Medicine Faculty of Medicine University of Lisbon, Av. Professor Egas Moniz Lisboa 1649-028 Portugal

## Abstract

The cadherin superfamily is a diverse and multifunctional group of proteins with extensive representation across genomes of phylogenetically distant species that is involved in cell–cell communication and adhesion. The mosquito *Anopheles gambiae* is an emerging model organism for the study of innate immunity and host–pathogen interactions, where the malaria parasite induces a profound rearrangement of the actin cytoskeleton at critical stages of infection. We have used bioinformatics tools to retrieve present sequence knowledge about the complete repertoire of cadherins in *A. gambiae* and compared it to that of the fruit fly, *Drosophila melanogaster*. In *A. gambiae*, we have identified 43 genes coding for cadherin extracellular domains that were re-annotated to 38 genes and represent an expansion of this gene family in comparison to other invertebrate organisms. The majority of *Drosophila* cadherins show a 1 : 1 *Anopheles* orthologue, but we have observed a remarkable expansion in some groups in *A. gambiae*, such as N-cadherins, that were recently shown to have a role in the olfactory system of the fruit fly. *In vivo* dsRNA silencing of overrepresented genes in *A. gambiae* and other genes showing expression at critical tissues for parasite infection will likely advance our understanding of the problems of host preference and host–pathogen interactions in this mosquito species.


**Comparative and Functional Genomics**
*Comp Funct Genom* 2005; **6**: 204–216.
Published online in Wiley InterScience (www.interscience.wiley.com). **DOI:** 10.1002/cfg.473

**Research Article**

### The cadherin superfamily in *Anopheles gambiae*: a comparative study with *Drosophila melanogaster*

Catarina Moita[1]*, Sérgio Simões[1†], Luís F. Moita[2], António Jacinto[1†] and Pedro Fernandes[1]

[1] *Instituto Gulbenkian de Ciência, Rua da Quinta Grande 6, Apartado 14, 2781-901 Oeiras, Portugal*
[2] *Center for Immunology and Inflammatory Diseases, Division of Rheumatology, Allergy and Immunology, Massachusetts General Hospital, 149 13th Street, Room 8301, Charlestown, MA 02129, USA*

**Correspondence to: Catarina Moita, Instituto Gulbenkian de Ciência, Rua da Quinta Grande 6, Apartado 14, 2781-901 Oeiras, Portugal. E-mail: cmoita@igc.gulbenkian.pt*

*† Current address: Institute of Molecular Medicine, Faculty of Medicine, University of Lisbon, Av. Professor Egas Moniz, 1649-028 Lisboa, Portugal..*

Received: 27 July 2004
Revised: 18 January 2005
Accepted: 8 March 2005

### Abstract

**The cadherin superfamily is a diverse and multifunctional group of proteins with extensive representation across genomes of phylogenetically distant species that is involved in cell–cell communication and adhesion. The mosquito *Anopheles gambiae* is an emerging model organism for the study of innate immunity and host–pathogen interactions, where the malaria parasite induces a profound rearrangement of the actin cytoskeleton at critical stages of infection. We have used bioinformatics tools to retrieve present sequence knowledge about the complete repertoire of cadherins in *A. gambiae* and compared it to that of the fruit fly, *Drosophila melanogaster*. In *A. gambiae*, we have identified 43 genes coding for cadherin extracellular domains that were re-annotated to 38 genes and represent an expansion of this gene family in comparison to other invertebrate organisms. The majority of *Drosophila* cadherins show a 1 : 1 *Anopheles* orthologue, but we have observed a remarkable expansion in some groups in *A. gambiae*, such as N-cadherins, that were recently shown to have a role in the olfactory system of the fruit fly. *In vivo* dsRNA silencing of overrepresented genes in *A. gambiae* and other genes showing expression at critical tissues for parasite infection will likely advance our understanding of the problems of host preference and host–pathogen interactions in this mosquito species. Copyright © 2005 John Wiley & Sons, Ltd.**

**Keywords:** **cell-adhesion; comparative genomics; *Anopheles gambiae*; *Drosophila melanogaster*; cadherin**

### Introduction

The cadherin superfamily is an extensive and multifunctional group of proteins present both in vertebrates and invertebrates. They are defined by the presence of an extracellular region commonly involved in calcium-dependent cell–cell adhesion, a transmembrane domain, and a cytoplasmic segment involved in cytoskeleton engagement (Nagafuchi and Takeichi, 1988; Takeichi *et al.*, 1988). The extracellular region includes a variable number of cadherin repeats (Figure 1), which contain the conserved motifs DRE, DXNDNAPXF and DXD (Oda *et al.*, 1994; Takeichi, 1990). Cadherins are often implicated in the specificity of cell–cell adhesion (Niessen and Gumbiner, 2002; Nose *et al.*, 1988), cell signalling (Frank and Kemler, 2002; Kovacs *et al.*, 2002; Noren *et al.*, 2001; Yagi and Takeichi, 2000), cell polarity (Chae *et al.*, 1999; Curtin *et al.*, 2003; Usui *et al.*, 1999), as well as in morphogenetic movements (Godt and Tepass, 1998; Togashi *et al.*, 2002) and tumour suppression (Perl *et al.*, 1998; Semb and Christofori, 1998).

Cadherins have been revised and generally categorized into several groups: classical (type I and II), desmosomal, protocadherins, Flamingo cadherins and a group of unique members that do not fit

Copyright © 2005 John Wiley & Sons, Ltd.

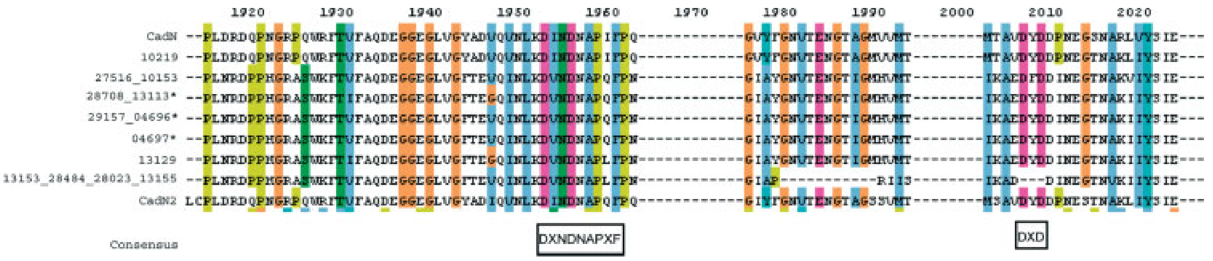

**Figure 1.** Multiple sequence alignment of cadherin domains in representative *D. melanogaster* and *A. gambiae* proteins using ClustalX. Conserved cadherin motifs are boxed into any of the previous groupings (Nollet *et al*., 2000; Tepass *et al*., 2000). Type I cadherins have five extracellular cadherin domains and a conserved His–Ala–Val (HAV) region in the first ectodomain (Blaschuk *et al*., 1990). Type II cadherins show a similar domain structure to type I cadherins, but do not have the HAV conserved motif (Tanihara *et al*., 1994). The desmosomal group is divided into two subgroups, desmocollins and desmogleins, which are present at desmosomal junctions (King *et al*., 1997). Protocadherins form the largest cadherin subgroup in mammals (Frank and Kemler, 2002). Most of the mammalian protocadherins are expressed in the central nervous system, and are enrolled in tissue morphogenesis and formation of neuronal pathways from early development (Redies, 2000; Shapiro and Colman, 1999; Yagi and Takeichi, 2000). The Flamingo subgroup is defined by the presence of seven-pass transmembrane regions instead of the single transmembranar domain of the other subgroups (Usui *et al*., 1999). Fat-type and Ret-like cadherins are examples of cadherin members that do not comply with the criteria of any of the subgroups described above.

The diversity of processes in which cadherins are involved has generated much interest in this family. Several members of the family have been extensively studied in model organisms, particularly *D. melanogaster* and *Caenorhabditis elegans*, whose complete repertoires were determined and compared (Hill *et al*., 2001).

Female *A. gambiae* mosquitoes, a vector of human malaria, require a vertebrate blood meal to complete their life cycle. *A. gambiae* is strongly anthropophilic but the molecular mechanisms that explain the recognition of the human host, mainly through olfactory cues, are still not well understood (Hallem *et al*., 2004). During the blood meal, mosquitoes can become infected with the agent of malaria — parasites of the genus *Plasmodium*.

*A. gambiae* is an emerging model organism with special relevance for the study of innate immunity and host–pathogen interactions (Christophides *et al*., 2002). The recent determination of its complete genome sequence (Holt *et al*., 2002; Mongin *et al*., 2004) offers an unprecedented opportunity for the identification of new factors involved in host preference and host–pathogen interactions that might determine the ability of this mosquito to transmit malaria. The comparison of a family of proteins known to be fundamental for cell–cell interactions and cell signalling in two insect dipteran species which have very different lifestyles might be an important contribution towards this goal and for a more global and deeper understanding of cadherin functions and evolution.

In this study, we have used bioinformatics methods to retrieve the complete repertoire of *A. gambiae* cadherins, to compare it to that of *D. melanogaster* and to use this information to identify members of this superfamily with possible relevance for the mosquito life cycle. This information is also relevant to understanding the cadherin superfamily and its evolution.

### Materials and methods

### Selection of protein sequences for the study

The study was based on a set of protein sequences from *D. melanogaster* (Table 1) and *A. gambiae* (Table 2) available in public databases with characteristic cadherin domains (known or predicted). This set was analysed and compared in the two species in study.

The identification of protein sequences presenting cadherin domains was performed by combining

Copyright © 2005 John Wiley & Sons, Ltd. 

**Table 1.** *D. melanogaster* cadherin genes

| Gene ID | Gene name | Genome location | Physical map Start | Stop | Strand | Features | Number of cadherin ectodomains assigned by SMART |
|---|---|---|---|---|---|---|---|
| CG6445 | cad74A | 3L 17.3 Mb | 17.316.630 | 17.328.408 | − | TM, SP | 13 |
| CG3389 | cad88C | 3R 10.5 Mb | 10.460.493 | 10.467.352 | + | TM, SA | 14 |
| CG4655/CG4509 | cad86C | 3R 6.7 Mb | 6.663.133<br>6.668.530 | 6.667.791<br>6.674.242 | + | TM | (2 + 3) 5 |
| CG10421 | cad96Cb | 3R 21.0 Mb | 21.040.762 | 21.043.289 | − | TM | 4 |
| CG3722 | shotgun | 2R 16.1 Mb | 16.094.146 | 16.100.703 | − | TM, SA | 7 |
| CG11895 | stan | 2R 5.8 Mb | 5.770.635 | 5.785.870 | + | Seven TM domain, SP | 8 |
| CG7749 | fat2 | 3L 19.9 Mb | 19.927.373 | 19.943.051 | + | TM, SP | 34 |
| CG31009 | cad99C | 3R 25.7 Mb | 25.660.692 | 25.669.791 | + | TM, SP | 11 |
| CG11059 | cals | 4 1.1 Mb | 1.134.646 | 1.142.866 | − | TM, SP | 2 |
| CG6977 | cad87A | 3R 7.7 Mb | 7.719.243 | 7.727.933 | − | TM | 14 |
| CG14900 | cad89D | 3R 12.3 Mb | 12.310.439 | 12.321.159 | − | TM, SA | 12 |
| CG10244 | cad96Ca | 3R 21.0 Mb | 21.013.946 | 21.024.660 | − | TM, SA | 1 |
| CG7527 | cadN2 | 2L 17.8 Mb | 17.764.688 | 17.790.687 | − | TM, SA | 6 |
| CG7100 | cadN | 2L 17.6 Mb | 17.627.949 | 17.717.604 | − | TM, SA | 16 |
| CG3352 | fat | 2L 4.2 Mb | 4.190.971 | 4.210.421 | − | TM, SP | 34 |
| CG17941 | dachsous | 2L 0.6 Mb | 641.686 | 716.633 | − | TM, SA | 27 |

SP, signal peptide; SA, signal anchor; TM, transmembrane region.

previous work on this family in *D. melanogaster* (Hill *et al.*, 2001), with assignments for this domain available in SUPERFAMILY (**http://supfam.mrc-lmb.cam.ac.uk/SUPERFAMILY/**) (Gough *et al.*, 2001). SUPERFAMILY is a database of identified domains within proteins of known structure using Hidden Markov Models (HMM), relying on the Structural Classification of Proteins (SCOP) database (Andreeva *et al.*, 2004; Murzin *et al.*, 1995). The SCOP database consists of a hierarchical classification of the domains of all proteins of known structure according to their evolutionary and structural relationships. The current SUPERFAMILY database (Madera *et al.*, 2004) uses versions 3.1 and 19.2a of the *D. melanogaster* and *A. gambiae* genomes, respectively, for the assignments to all predicted proteins in these organisms.

The sequences selected were those that matched for cadherin domains by HMM with an expectation value score below 0.001. *D. melanogaster* sequences fulfilling the above criteria were retrieved from FlyBase (**http://flybase.bio.indiana.edu/**) and *A. gambiae* sequences were retrieved from Ensembl (**http://www.ensembl.org**).

The designations used in this report for the sequences considered are the terms attributed by the genome sequencing projects, or those previously given by researchers.

### Identification of other domains, signal peptides and transmembrane helices

The protein sequences identified as having one or more cadherin domains were inspected for other features and domains, using the following servers: (a) InterPro metaserver (Zdobnov and Apweiler, 2001), which includes several databases and scanning methods to check additional protein domains; (b) SignalP 3.0 server (Dyrlov Bendtsen *et al.*, 2004; Nielsen *et al.*, 1999), with default options for eukaryotes to identify signal peptide sequences; (c) TMHMM server (Krogh *et al.*, 2001), with default parameters for transmembrane helices, intracellular and extracellular region prediction; (d) SMART server (Letunic *et al.*, 2002; Schultz *et al.*, 1998), to perform a quick domain inspection and to check InterPro matches.

### Revision of gene predictions

The predicted protein sequences in the referred species were matched against experimental sets, in order to check for possible corrections. The

Copyright © 2005 John Wiley & Sons, Ltd. 

**Table 2.** *A. gambiae* cadherin genes and proposed mergers

| Gene ID | Gene name | Genome location | Physical map: Start | Physical map: Stop | Physical map: Strand | Features | Number of cadherin ectodomains assigned by SMART |
|---|---|---|---|---|---|---|---|
| 05 677* | | 2L 43.7 Mb | 43.727.328 | 43.731.215 | + | | 4 |
| 22 839 | | 3R 27.8 Mb | 27.836.031 | 27.837.090 | + | | Cadherin Cytoplasmic_term (Pfam) |
| 10 640 | | 3R 40.2 Mb | 40.163.652 | 40.233.156 | − | TM | 6 |
| 07 491 | | 3R 41.7 Mb | 41.652.745 | 41.659.254 | − | | 1 |
| 22 442 | | 3R 41.7 Mb | 41.652.332 | 41.652.699 | − | | Cadherin Cytoplasmic_term (Pfam) |
| 03 678* | | 3R 40.9 Mb | 40.940.012 | 40.944.886 | + | TM | 6 |
| 01 449 | | 3R 42.3 Mb | 42.264.834 | 42.465.931 | + | TM | 17 |
| 23 672/16 780 | | X 2.5 Mb | 2.466.406 | 2.468.735 | + | SP | 5 |
| | | | 2.468.796 | 2.475.772 | | TM | |
| 15 646 | | 2R 6.5 Mb | 6.519.174 | 6.525.698 | + | TM, SA | 13 |
| 18 160* | | 2R 19.1 Mb | 19.076.395 | 19.077.321 | − | TM | 10 |
| 18 163 | | 2R 19.1 Mb | 19.133.372 | 19.135.189 | − | SP | 1 |
| 13 734* | | 2R 28.1 Mb | 28.059.369 | 28.066.249 | + | TM | 8 |
| 08 806* | | 2R 42.1 Mb | 42.099.204 | 42.136.342 | − | TM | 13 |
| 09 438* | | 2R 42.4 Mb | 42.401.168 | 42.402.572 | + | TM | 4 |
| 08 765 | | 2R 52.8 Mb | 52.801.372 | 52.808.264 | − | TM | 11 |
| 22 133 | | UNKN 17.3 Mb | 17.296.450 | 17.297.694 | + | | 2 |
| 09 564 | | UNKN 42.9 Mb | 42.851.852 | 42.892.216 | + | | 5 |
| 01 796 | | UNKN 57.3 Mb | 57.254.116 | 57.264.908 | + | SP | 9 |
| 12 071* | | UNKN 57.3 Mb | 57.315.015 | 57.316.304 | + | | 3 |
| 12 062* | | UNKN 57.3 Mb | 57.347.922 | 57.370.069 | + | TM, SA | 14 |
| 01 056 | GPRstn | 2L 3.9 Mb | 3.930.218 | 3.941.726 | − | 7 Pass TM domain, SP | 8 |
| 08 654† | | 2L 42.2 Mb | 42.178.354 | 42.204.792 | + | | 2 |
| 15 226* | | 2L 26.4 Mb | 26.437.824 | 26.439.233 | + | SP | |
| 18 042 | | 2L 28.1 Mb | 28.080.974 | 28.093.877 | − | TM, SP | 13 |
| 24 272/04 442*‡ | | 2L 36.8 Mb | 36.758.209 | 36.765.031/ | + | | 6 |
| | | | 36.796.892 | 36.799.716 | | | |
| 19 148* | | 2L 36.7 Mb | 36.742.085 | 36.816.952 | − | SP | 5 |
| 07 172* | | 2L 43.8 Mb | 43.803.194 | 43.806.634 | − | | 5 |
| 16 988 | | 2L 43.7 Mb | 43.737.820 | 43.740.501 | + | | 7 |
| 05 443 | | 3R 2.9 Mb | 2.856.125 | 2.897.703 | − | TM | 34 |
| 22 629 | | 3R 2.7 Mb | 2.670.906 | 2.672.729 | − | SP | 5 |
| 07 504 | | 3R 2.6 Mb | 2.612.104 | 2.619.915 | − | TM | 22 |
| 22 750/10 624* | | 3R 40.1 Mb/3R 40.2 Mb | 40.129.467 | 40.129.908 | + | | 6 |
| | | | 40.155.431 | 40.160.309 | | | |
| 24 523/03 677* | | 3R 40.7 Mb/3R 40.6 Mb | 40.659.508 | 40.659.994 | − | TM | 6 |
| | | | 40.639.251 | 40.644.478 | | | |
| 24 401/07 664 | | 3R 41.9 Mb | 41.918.478 | 41.918.908 | − | TM | 6 |
| | | | 41.895.947 | 41.901.977 | | | |
| 23 811/22 581/ 24 896/10 666 | | 3R 40.4 Mb | 40.443.445 | 40.453.729 | − | TM | 7 |
| | | | 40.427.979 | 40.437.650 | | | |
| | | | 40.427.082 | 40.427.939 | | | |
| | | | 40.424.415 | 40.426.950 | | | |
| 24 301/19 805*/ 19 810‡ | | 3L 25.9 Mb | 25.943.285 | 25.946.595 | − | TM | 11 |

Copyright © 2005 John Wiley & Sons, Ltd. 

**Table 2.** Continued

| Gene ID | Gene name | Genome location | Physical map Start | Stop | Strand | Features | Number of cadherin ectodomains assigned by SMART |
|---|---|---|---|---|---|---|---|
| | | | 25.908.350 | 25.911.221 | | | |
| | | | 25.874.662 | 25.876.951 | | | |
| 16655* | | 3L 30.1 Mb | 30.109.914 | 30.112.521 | + | TM, SP | 1 |

The Ensembl designation for genes was abbreviated by omitting the prefix ENSANGG000000. In the case of existence of different splice variants, the longest sequence was considered.
* Experimentally determined sequence is the longest.
† Gene predicted by the previous Ensembl automatic annotation of the *A. gambiae* genome sequence.
‡ Proposed merger not confirmed.
SP, signal peptide; SA, signal anchor; TM, transmembrane region.

sets used were the non-redundant public protein database UniProt (Apweiler *et al*., 2004) and, in the case of *Drosophila*, a second set was obtained by screening a library of more than 9000 cDNAs (**http://www.fruitfly.org/sequence/dlcDNA.shtml**) using FASTA (Pearson and Lipman, 1988). The program implementation used for the first set was fasta3, available at the European Bioinformatics Institute (EBI) website with default parameters (**http://www.ebi.ac.uk/fasta33/**), an upper expectation value of 0.001 and sequence identity higher than 50%. For the cDNA database, a locally installed version of FASTA within the GCG (Wisconsin) package version 10.0 was used, with default parameters and an upper expectation value of 0.05. For this second database, the FASTA algorithm showed undesired sensitivity to intron presence, therefore the Smith–Waterman algorithm (EMBOSS-water implementation) and SIM4 (Florea *et al*., 1998) were used, both with default parameters. Although no new possible corrections were detected by this approach for *D. melanogaster*, in the case of *A. gambiae* we detected several matches in the UniProt database that were identical and extended the predicted protein sequences.

### EST information

Whenever possible, we considered EST sequence information, in order to confirm the structure of the predicted *Anopheles* genes. In two cases, the ESTs associated to the gene annotation extended their predicted ends. In other cases, the ESTs have supported the predictions. These aspects are summarized in Table 3.

### Multiple alignments

The cadherin sequences that were identified and re-annotated according to the previously described procedures were aligned using ClustalX 1.83 (Thompson *et al*., 1997), and a tree representation (Figure 2) was generated from the multiple alignment by the same program using the neighbour-joining method. In these alignments, we have included the longest sequences (either the predicted or the experimentally obtained fragment); proteins known to have splice variants were represented by the longest isoform. Sequences shorter than 350 amino acids in length were not considered in order to not affect the alignment (see Table 4).

### Results and discussion

### Merged genes

In this study, the Ensembl designation for *Anopheles* genes and proteins was abbreviated by omitting the prefixes ENSANGG000000 and ENSANGP-000000, respectively.

In *A. gambiae*, by inspecting the positions of the predicted sequences (Figure 3), seven sets of predicted cadherin proteins were identified whose gene sequences are adjacent on their respective chromosomes. Merging the adjacent predicted genes resulted in gene products with more cadherin

Copyright © 2005 John Wiley & Sons, Ltd. 

**Table 3.** EST information for *Anopheles* genes. The Ensembl designations for genes and ESTs are abbreviated by omitting the prefix ENSANGG000000 and ENSANGESTG00000, respectively

| Gene | EST | Notes |
|---|---|---|
| 04 442 | 351 441 | The sequences are closely downstream to the gene, which suggests that the predicted gene sequence is incomplete as it only has four cadherin domains, one EGF and one LamG domain. |
| | 351 449 | |
| 05 677 | 349 836 | Matches an initial portion of the first predicted exon of one of the possible transcripts of this gene (ENSANGT00000028342), also extending it upstream, allowing the identification of an extra cadherin domain. |
| 01 449 | 345 294 | Confirmation of the gene structure predicted so far. |
| | 345 316 | |
| | 345 327 | |
| 08 654 | 346 815 | Confirmation of the gene structure predicted in the previous Ensembl release. |
| 16 655 | 356 396 | Confirmation of the gene structure predicted so far. |
| | 356 397 | |
| 13 734 | 344 926 | Matches the last exon predicted, but also extends it in the 3′ end. |
| 09 564 | 348 631 | Confirmation of the gene structure predicted so far |
| 12 062 | 345 399 | Confirmation of the gene structure predicted so far |
| 07 504 | 354 730 | Confirmation of the gene structure predicted so far |
| 15 226 | 350 926 | Matches exactly the prediction in all its extension |

**Table 4.** Sequences shorter than 350 amino acids (these were not considered in the comparative analysis)

| Gene | Protein | Location |
|---|---|---|
| 07 491 | 09 980 | 41.7 3R |
| 12 071 | 14 560 | Unknown |
| 15 226 | 17 715 | 26.4 2L |
| 18 163 | 20 652 | 19.1 2R |
| 22 133 | 28 873 | Unknown |
| 22 442 | 25 527 | 41.7 3R |
| 22 839 | 27 510 | 27.8 3R |

domains and, in some cases, other domains and transmembrane regions.

In five cases, the proposed union of genes is confirmed by experimental sequence matching found in UniProt database; this is the case for the merging of four genes 23 811, 22 581, 24 896 and 10 666, as well as for 23 672 + 16 780, 22 750 + 10 624, 24 523 + 03 677 and 24 401 + 07 664. Two other possible merging sets (24 272 + 04 442 and 24 301 + 19 805 + 19 810) are proposed, based only on the gene proximity and congruence of the domain architecture of the resulting putative protein. Figure 4 shows the domain architecture of the *Anopheles* cadherin repertoire with the proposed gene mergers.

### Cytoplasmic domains

The *D. melanogaster* cadherin genes encode cytoplasmic domains of 43–968 amino acid residues, in agreement with Hill *et al.* (2001). For *A. gambiae*, taking into account only those sequences for which a transmembrane region was predicted (and considering that some might be incomplete at their 3′ ends), the cytoplasmic regions vary (ca. 38–539 residues).

### Similar and unique cadherins in *D. melanogaster* and *A. gambiae*

The domain architectures of vertebrate and invertebrate cadherins show several differences (Oda and Tsukita, 1999). The classification adopted in this report follows, to some extent, previous studies (Angst *et al.*, 2001; Nollet *et al.*, 2000; Tepass *et al.*, 2000). For simplification and discussion of organization, cadherins will be considered ‘classical’ (showing a conserved cytoplasmic domain that can bind to catenins) or ‘non-classical’, and this second category includes sub-groups of ‘Fat-like’, ‘Flamingo’, ‘Ret-like’ and ‘Other’. The *Drosophila* cadherin Dachsous is included in the ‘Fat-like’ sub-group as it has a large number of cadherin ectodomains but, as mentioned in Gooding *et al.* (2004), shows sequence similarity to the cytoplasmic $\beta$-catenin-binding domain of classical vertebrate cadherins.

### Classical

*cadN/01 449 and other possible N-like cadherins*
The *Drosophila* protein sequence CadN matches

Copyright © 2005 John Wiley & Sons, Ltd.

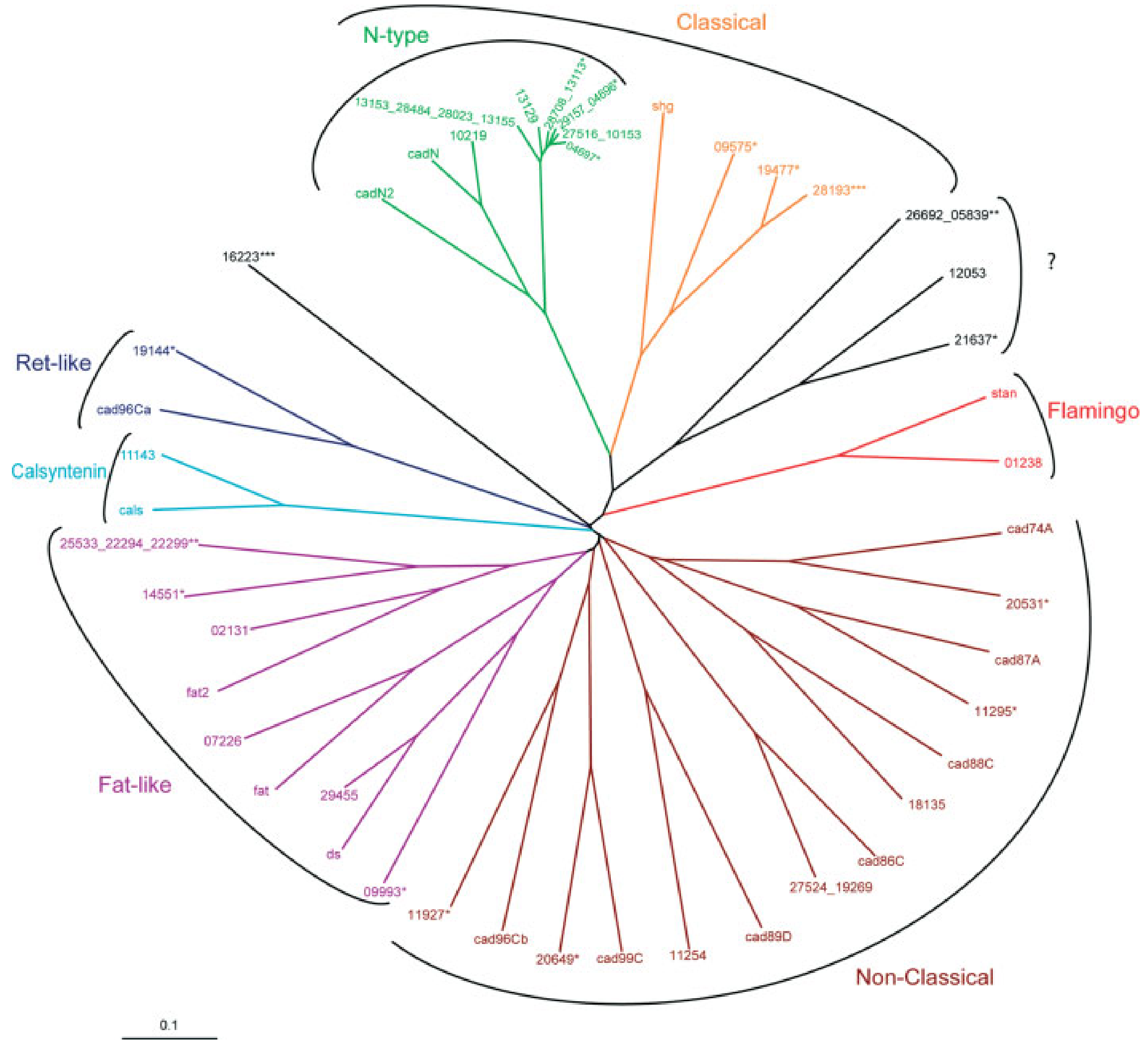

**Figure 2.** Radial tree representation of the cadherin proteins in *A. gambiae* and *D. melanogaster*. Sequences were aligned using ClustalX and the tree derived with the neighbour-joining algorithm. *Drosophila* sequences are referred to by their gene name. *Anopheles* sequences are referred to by their Ensembl entry, omitting the prefix ENSANGP000000. Proteins known to have splice variants are represented by the longest available sequence. Sequences shorter than 350 amino acids in length were omitted. *Experimental fragment is the longest available sequence; **Proposed merger not validated experimentally; ***Sequence extended by overlapping EST

with a FASTA e-value of zero to two transcripts of this *Anopheles* gene (proteins 10 175 and 10 219) with 87% sequence identity, which demonstrates the high conservation of these sequences in all their extension. Besides this mosquito gene, this species also has six other genes that encode for proteins with similar domain organization (with minor differences in terms of cadherin repeat number), to which this sequence and the fruit fly CadN align by BLAST with an e-value of zero. All the sequences form a cluster (Figure 2) suggesting that this subgroup has experienced an expansion in *Anopheles*. All of these mosquito genes are localized on the same chromosome (3R) and adjacent to each other (Figure 3).

The cadN gene product has putative orthologues in other species by reciprocal BLAST analysis, such as *C. elegans* W02B9.1, *C. briggsae* CBG07964, and novel predictions in the zebrafish, *Danio rerio*, (ENSDARG00000001983); chicken (ENSGALG00000004630); and the pufferfish, *Fugu rubripes,* (SINFRUG00000151656).

Copyright © 2005 John Wiley & Sons, Ltd. 

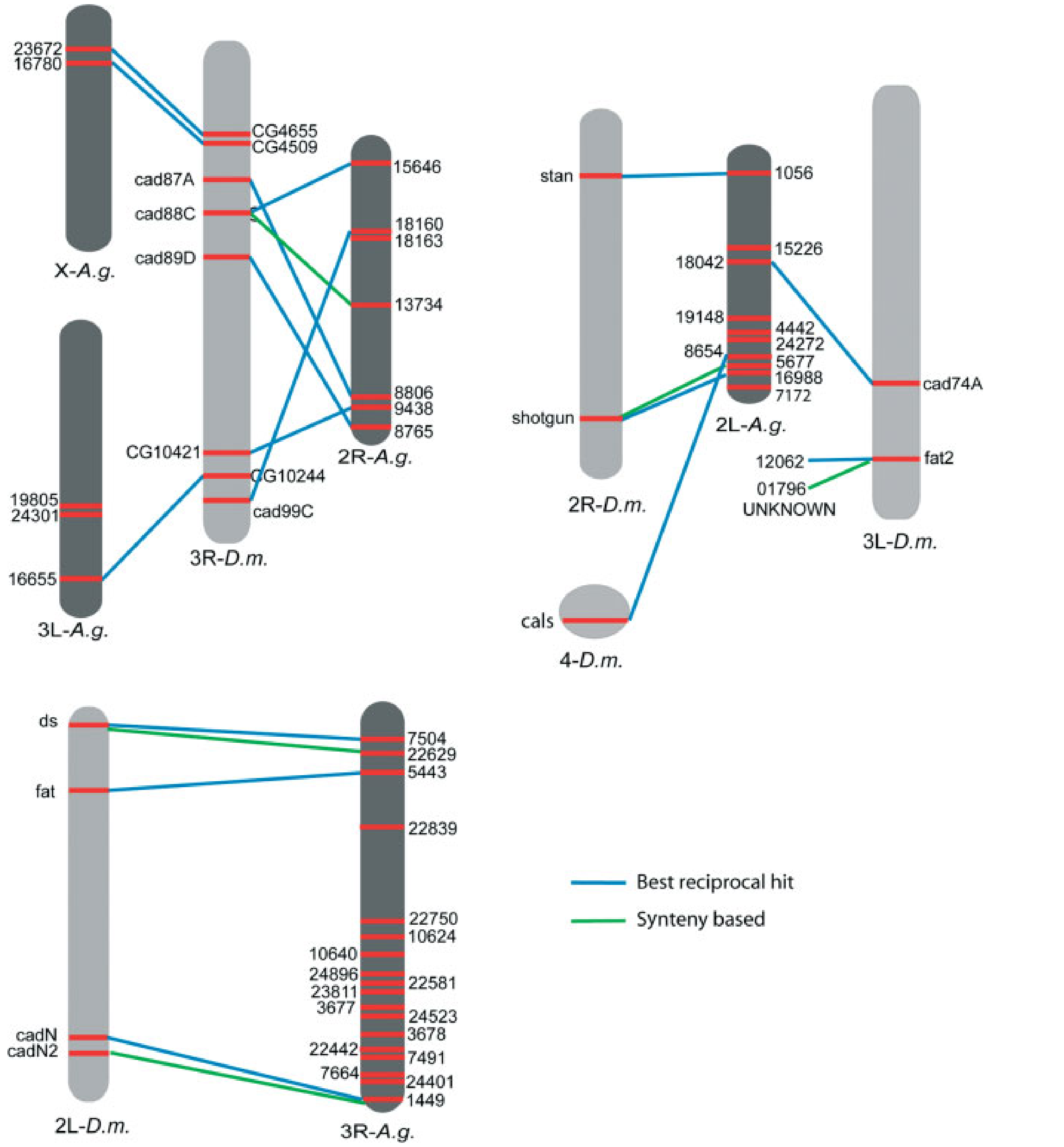

**Figure 3.** Chromosomal locations of *D. melanogaster* and *A. gambiae* cadherin genes. Putative orthologues are indicated. Lines are coloured blue for orthology based on BLAST best reciprocal hit, and green for synteny around the best reciprocal match

The cadN gene is reported to be expressed in neurons, also regulating neuronal morphogenesis (Iwai *et al.*, 1997) and is proposed to be involved in synaptic target specificity (Lee *et al.*, 2001).

*A. gambiae* is highly anthropophilic and finds human hosts largely through olfactory cues (Hallem *et al.*, 2004). Recently, N-cadherins were implicated in *D. melanogaster* olfaction (Hummel and Zipursky, 2004; Zhu and Luo, 2004). In spite of the eight possible alternatively spliced isoforms for N-cadherin, the expression of one isoform is sufficient to rescue all affected phenotypes (Zhu and Luo, 2004).

Copyright © 2005 John Wiley & Sons, Ltd. 

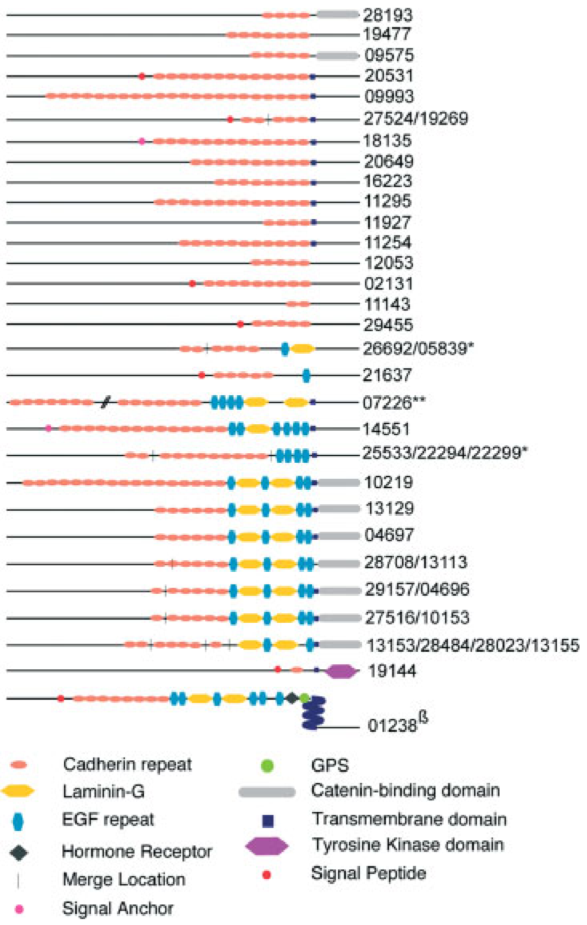

**Figure 4.** Schematic representation of the domain organization of the *Anopheles* cadherin repertoire. Proteins known to have splice variants are represented only once, by the longest sequence available. Sequences shorter than 350 amino acids in length are not represented. *Proposed merger not validated experimentally; **07226 sequence contains a total of 34 cadherin repeats, 20 of which were omitted at the double slash location to simplify the representation; $^{\beta}$01 238 sequence has a seven-pass transmembrane region

*Shotgun (DE-cadherin) Anopheles* 19 477 is a putative orthologue for Shotgun (*Drosophila* E-cadherin), but the protein fragment currently available in public databases shows differences in terms of domain architecture, viz. the assignment of an EGF domain by SMART and the non-detection, to date, of a transmembrane region or a classical cadherin cytoplasmic segment. Moreover, it is possible to distinguish a synteny-based hit with 28 193, again with several differences at the domain architecture level, such as the number of cadherin extracellular repeats. The gene product 09 575 also clusters with these sequences (Figure 2).

The shotgun gene is a classical epithelial cadherin, expressed in a broad range of tissues, and it has been shown to be required for tissue integrity, oogenesis and cell rearrangements during morphogenesis (Haag *et al.*, 1999; Oda *et al.*, 1997; Tepass *et al.*, 1996).

It should be stressed that we are considering *Anopheles* sequences that are probably incomplete, and future experimental work should therefore help to clarify the gene architecture and protein domain organization of these sequences.

### Non-classical

*Fat-like* fat/05 443, fat2/12 062 Of the possible orthologues between the *Drosophila* and *Anopheles* sequences, Fat and 07 226 show 60% identity in Smith–Waterman local pairwise alignment, and Fat2 and 14 551 show 47% sequence identity. Nevertheless, for Fat2, besides the possibility of distinguishing a synteny-based orthologue (02 131), one of the proposed merging genes also clusters with these sequences (Figure 2). This possible union is not yet confirmed, but the domain structure of the putative product shows some similarities with Fat-like sequences.

Particularly in the case of the Fat and 07 226 proteins, there is a remarkable similarity between the two sequences, even in the cytoplasmic region; both of them have BLAST sequence alignments with an e-value of zero to several fat-like proteins in other species, such as rat Fat, Fat 2 and Fat 3, human Fat, mouse Fat 1 cadherin and zebrafish Fat, all of which have similar domain structures.

The *Drosophila* fat gene controls cell growth (Agrawal *et al.*, 1995; Garoia *et al.*, 2000) by acting as a tumour-suppressor gene (Bryant *et al.*, 1993) and it is involved in planar polarity (Casal *et al.*, 2002; Fanto *et al.*, 2003; Rawls *et al.*, 2002). It has been shown that *fat2* is the true orthologue of the vertebrate fat-like cadherins (Tepass *et al.*, 2000), and more recently Castillejo-Lopez *et al.* (2004) have reported its involvement in the formation of tubular organs.

dachsous/07 504 In the case of *Drosophila* dachsous, there is an *Anopheles* sequence presenting a best reciprocal hit (09 993), which demonstrates

Copyright © 2005 John Wiley & Sons, Ltd. 

58% sequence identity and similar domain organization. Additionally, dachsous has a possible synteny-based orthologue in 29 455, but its predicted protein domain organization is substantially different. Considering the close proximity of the two *Anopheles* genes, and the domain organization of the products coded, it is possible that they should be merged but, as we did not find any experimental evidence to confirm this, we have considered them separately.

The dachsous gene is involved in the control of imaginal disc morphogenesis (Clark *et al.*, 1995); it is shown to have a role in planar polarity (Casal *et al.*, 2002; Eaton, 2003), as well as in regulating dorsal–ventral signalling in the *Drosophila* eye (Rawls *et al.*, 2002).

*Flamingo* flamingo/01 056 (GPRstn) As in *D. melanogaster* and *C. elegans* (Hill *et al.*, 2001), *A. gambiae* has one seven-helix transmembrane cadherin, which leads us to consider Flamingo and GPRstn as orthologues. The protein sequences match with a FASTA e-value of zero with 66% identity, presenting the same number of cadherin domains, as well as of EGF, LamG, GPS (G-protein-coupled receptor proteolytic site) and HMR (hormone receptor) domains. Also, their extracellular and cytoplasmic regions are of similar length and conservation.

In BLAST searches, the Flamingo and 01 238 proteins produce sequence matches with e-values of zero, to several proteins containing a seven-helix transmembrane region, viz: mouse mFmi1, MEGF2, CELSR1, CELSR3; rat CELSR2, CELSR3; human CELSR1, CELSR2, CELSR3 and CLR1; *C. elegans* FMI-1; and a hypothetical protein of *C. briggsae* (CBG09454), all of which have high sequence similarity to each other; and a *D. rerio* sequence (CAE30365). The extracellular domain organization of the above sequences is very similar, except for the *D. rerio* entry which does not have cadherin domains based on the current domain assignments. However, in terms of overall sequence observation, there is some degree of conservation within vertebrate and invertebrate groups, but not between them.

Flamingo is known to be involved in planar polarity (Chae *et al.*, 1999; Usui *et al.*, 1999) and, more recently, to be engaged in neuronal differentiation, dendritic development (Sweeney *et al.*, 2002) and target interactions in the *Drosophila* visual system (Lee *et al.*, 2003; Senti *et al.*, 2003).

*Ret-like* cad96Ca/16 655 In the case of Cad96-Ca, *Anopheles* presents a possible orthologue, 19 144 (coded by gene 16 655), which encodes a signal peptide, a cadherin domain, a transmembrane region and a cytoplasmic segment with a tyrosine kinase domain, similarly to the *Drosophila* sequence. The two sequences have 53% sequence identity in Smith–Waterman pairwise alignment, showing high conservation, particularly in the cytoplasmic region.

*Other* CG4655_CG4509 (cad86C)/23 672_16 780 This *Drosophila* possible merger proposed by Hill *et al.* (2001) is still considered as such, as no new experimental evidence supports the existence of a unique gene. Nevertheless, the identification of a similar possible merger in *Anopheles* (and its confirmation by a match in UniProt), 27 524_19 269, with 61% sequence identity from Smith–Waterman local pairwise alignment to the *Drosophila* union, seems to support the likeliness of the proposed arrangement. However, it is important to remember that the *Drosophila* genome annotation was used to annotate *Anopheles* and this might influence to some extent the gene structure proposed for *Anopheles* genes.

cad74A/18 042 The predicted gene products have similar length, extracellular regions and have an equal number of cadherin domains, matching with a FASTA e-value of zero and 55% identity.

cad87A/08 806 The two protein sequences have a Smith–Waterman identity of 58% and an equal number of cadherin domains, as well as a high conservation in their extracellular region. However, the current *Anopheles* sequence is shorter by 112 amino acids.

cad89D/08 765 Similarly to the observations for Cad87A, the proteins encoded by these genes show considerable similarity in their extracellular domains, with 41% sequence identity. As before, the *Anopheles* sequence is shorter (1821 amino acids, whereas Cad89D has 2240 residues) and no signal peptide has been identified.

Copyright © 2005 John Wiley & Sons, Ltd. 

cad99C/18 160 The protein sequences coded by these genes match with a FASTA e-value of zero and have 58% identity. Similarly to what was reported for Cad89D, the *Anopheles* fragment is smaller (by 98 amino acids) and no signal peptide has been identified so far.

cad96Cb/09 438 In the case of Cad96Cb, there is an *Anopheles* sequence representing a best reciprocal hit (11 927) which demonstrates sequence similarity and similar domain organization. A Smith–Waterman pairwise alignment shows 39% identity between the sequences.

cad88C/15 646 The *Anopheles* gene 15 646 is a putative orthologue of the fruit fly cad88C. However, the mosquito gene product has differences in terms of the number of cadherin repeats and sequence length (121 amino acids shorter). The protein sequences are 49% identical as shown by pairwise alignment.

calsyntenin (cals)/08 654 The Cals protein has a best reciprocal hit with *Anopheles* 11 143 protein, coded for by gene 08 654. The protein sequences show 59% identity by pairwise local alignment. The current *Anopheles* fragment in SwissProt (Q7QIW3) does not have a transmembrane region or signal peptide predicted. By best BLAST reciprocal hit, Cals has possible orthologues with other invertebrate and vertebrate species: B0034.3 from *C. elegans*, CBG02547 of *C. briggsae*, CLSTN2 from *Homo sapiens*, mouse Clstn1, Q7ZTX9 from *D. rerio*, and novel predictions from rat (ENSRNOG00000016398), chicken (ENSGALG00000005310) and *F. rubripes* (SINFRUG00000127288).

Cals is reported to be involved in synaptic transmission by binding synaptic $Ca^{2+}$ with its cytoplasmic domain (Vogt *et al.*, 2001).

#### Remaining cadherin repertoire

The remaining *Anopheles* sequences, to date, appear to have no remarkable similarity or possible *Drosophila* orthologues, beyond the fact of all having one or more cadherin domains.

### Conclusions

Cadherin ectodomains are distributed in the coded products of 17 *D. melanogaster* and 43 *A. gambiae* putative genes. These facts suggest an expansion of this protein family in *A. gambiae*. We propose seven possible gene mergers for *Anopheles* based on chromosome location analysis and neighbourhood inspection. From these, five were confirmed by sequence matches in public databases. *Anopheles* should now be considered to have 38 cadherin genes. If two additional unions are confirmed by future sequence data, a further reduction to 36 genes should then be considered.

Our chromosome localizations of the cadherin genes orthologous between *D. melanogaster* and *A. gambiae* (Figure 3) are in general agreement with the results reported by Zdobnov *et al.* (2002), in which the correspondence between chromosomes of the two species using 1 : 1 orthologues and microsynteny blocks was analysed. Specifically, chromosomal arm 2L of *Drosophila* is conserved relative to the *Anopheles* 3R arm, the same being the case for Dm3R and Ag2R; the *Anopheles* 2L chromosome hosts the majority of the *Drosophila* 2R and 3L orthologues. The only exceptions seem to be Cad96Ca and Cad86C and their respective orthologues. Moreover, the existence of several 1 : 1 orthologues is a promising contribution for subsequent work in functional genomics in the two species.

Among the identified genes, the group of N-cadherins is of particular interest because it has been dramatically expanded in *A. gambiae*. In *Drosophila*, there are two genes coding for this type of protein (one of which has eight possible transcripts), but in *Anopheles* it is possible to identify seven genes (one has four different possible transcripts).

The present study indicates that in the future, both experimental and theoretical work will be needed in order to confirm possible gene unions, as well as their 5′ and 3′ ends, as the majority of sequences in public databases are still incomplete.

The elucidation of the patterns of tissue expression of cadherins in *A. gambiae* should guide the selection of candidates for further work in the problem of host preference and host–pathogen interactions. The possibility of *in vivo* gene silencing by RNA interference provides a powerful approach to

Copyright © 2005 John Wiley & Sons, Ltd.

test the role of such candidates in these areas of intense study of the major vector of human malaria.

### Acknowledgements

We thank Hernan J. Dopazo, Luciano Milanesi and Maria Mota for their critical evaluation of the manuscript.

### References

Agrawal N, Joshi S, Kango M, *et al.* 1995. Epithelial hyperplasia of imaginal discs induced by mutations in *Drosophila* tumour suppressor genes: growth and pattern formation in genetic mosaics. *Dev Biol* **169**: 387–398.

Andreeva A, Howorth D, Brenner SE, *et al.* 2004. SCOP database in 2004: refinements integrate structure and sequence family data. *Nucleic Acids Res* **32**: (database issue): D226–229.

Angst BD, Marcozzi C, Magee AI. 2001. The cadherin superfamily: diversity in form and function. *J Cell Sci* **114**: 629–641.

Apweiler R, Bairoch A, Wu CH, *et al.* 2004. UniProt: the Universal Protein knowledgebase. *Nucleic Acids Res* **32**: (database issue): D115–119.

Blaschuk OW, Sullivan R, David S, Pouliot Y. 1990. Identification of a cadherin cell adhesion recognition sequence. *Dev Biol* **139**: 227–229.

Bryant PJ, Watson KL, Justice RW, Woods DF. 1993. Tumour suppressor genes encoding proteins required for cell interactions and signal transduction in *Drosophila*. *Development* (**suppl**): 239–249.

Casal J, Struhl G, Lawrence PA. 2002. Developmental compartments and planar polarity in *Drosophila*. *Curr Biol* **12**: 1189–1198.

Chae J, Kim MJ, Goo JH, *et al.* 1999. The *Drosophila* tissue polarity gene starry night encodes a member of the protocadherin family. *Development* **126**: 5421–5429.

Christophides GK, Zdobnov E, Barillas-Mury C, *et al.* 2002. Immunity-related genes and gene families in *Anopheles gambiae*. *Science* **298**: 159–165.

Clark HF, Brentrup D, Schneitz K, *et al.* 1995. Dachsous encodes a member of the cadherin superfamily that controls imaginal disc morphogenesis in *Drosophila*. *Genes Dev* **9**: 1530–1542.

Curtin JA, Quint E, Tsipouri V, *et al.* 2003. Mutation of Celsr1 disrupts planar polarity of inner ear hair cells and causes severe neural tube defects in the mouse. *Curr Biol* **13**: 1129–1133.

Dyrlov Bendtsen J, Nielsen H, Von Heijne G, Brunak S. 2004. Improved prediction of signal peptides: SignalP 3.0. *J Mol Biol* **340**: 783–795.

Eaton S. 2003. Cell biology of planar polarity transmission in the *Drosophila* wing. *Mech Dev* **120**: 1257–1264.

Fanto M, Clayton L, Meredith J, *et al.* 2003. The tumour-suppressor and cell adhesion molecule Fat controls planar polarity via physical interactions with atrophin, a transcriptional co-repressor. *Development* **130**: 763–774.

Florea L, Hartzell G, Zhang Z, *et al.* 1998. A computer program for aligning a cDNA sequence with a genomic DNA sequence. *Genome Res* **8**: 967–974.

Frank M, Kemler R. 2002. Protocadherins. *Curr Opin Cell Biol* **14**: 557–562.

Garoia F, Guerra D, Pezzoli MC, *et al.* 2000. Cell behaviour of *Drosophila* fat cadherin mutations in wing development. *Mech Dev* **94**: 95–109.

Godt D, Tepass U. 1998. *Drosophila* oocyte localization is mediated by differential cadherin-based adhesion. *Nature* **395**: 387–391.

Gough J, Karplus K, Hughey R, Chothia C. 2001. Assignment of homology to genome sequences using a library of hidden Markov models that represent all proteins of known structure. *J Mol Biol* **313**: 903–919.

Haag TA, Haag NP, Lekven AC, Hartenstein V. 1999. The role of cell adhesion molecules in *Drosophila heart* morphogenesis: faint sausage, shotgun/DE-cadherin, and laminin A are required for discrete stages in heart development. *Dev Biol* **208**: 56–69.

Hallem EA, Nicole Fox A, Zwiebel LJ, Carlson JR. 2004. Olfaction: mosquito receptor for human-sweat odorant. *Nature* **427**: 212–213.

Hill E, Broadbent ID, Chothia C, Pettitt J. 2001. Cadherin superfamily proteins in *Caenorhabditis elegans* and *Drosophila melanogaster*. *J Mol Biol* **305**: 1011–1024.

Holt RA, Subramanian GM, Halpern A, *et al.* 2002. The genome sequence of the malaria mosquito *Anopheles gambiae*. *Science* **298**: 129–149.

Hummel T, Zipursky SL. 2004. Afferent induction of olfactory glomeruli requires N-cadherin. *Neuron* **42**: 77–88.

Iwai Y, Usui T, Hirano S, *et al.* 1997. Axon patterning requires DN-cadherin, a novel neuronal adhesion receptor, in the *Drosophila* embryonic CNS. *Neuron* **19**: 77–89.

King IA, Angst BD, Hunt DM, *et al.* 1997. Hierarchical expression of desmosomal cadherins during stratified epithelial morphogenesis in the mouse. *Differentiation* **62**: 83–96.

Kovacs EM, Ali RG, McCormack AJ, Yap AS. 2002 E-cadherin homophilic ligation directly signals through Rac and phosphatidylinositol 3-kinase to regulate adhesive contacts.. *J Biol Chem* **277**: 6708–6718.

Krogh A, Larsson B, von Heijne G, Sonnhammer EL. 2001. Predicting transmembrane protein topology with a hidden Markov model: application to complete genomes. *J Mol Biol* **305**: 567–580.

Lee CH, Herman T, Clandinin TR, Lee R, Zipursky SL. 2001. N-cadherin regulates target specificity in the *Drosophila* visual system. *Neuron* **30**: 437–450.

Lee RC, Clandinin TR, Lee CH, *et al.* 2003. The protocadherin Flamingo is required for axon target selection in the *Drosophila* visual system. *Nat Neurosci* **6**: 557–563.

Letunic I, Goodstadt L, Dickens NJ, *et al.* 2002. Recent improvements to the SMART domain-based sequence annotation resource. *Nucleic Acids Res* **30**: 242–244.

Madera M, Vogel C, Kummerfeld SK, Chothia C, Gough J. 2004. The SUPERFAMILY database in 2004: additions and improvements. *Nucleic Acids Res* **32**: (database issue): D235–239.

Mongin E, Louis C, Holt RA, Birney E, Collins FH. 2004. The *Anopheles gambiae* genome: an update. *Trends Parasitol* **20**: 49–52.

Murzin AG, Brenner SE, Hubbard T, Chothia C. 1995. SCOP: a structural classification of proteins database for the investigation of sequences and structures. *J Mol Biol* **247**: 536–540.

Nagafuchi A, Takeichi M. 1988. Cell binding function of E-cadherin is regulated by the cytoplasmic domain. *EMBO J* **7**: 3679–3684.

Copyright © 2005 John Wiley & Sons, Ltd. 

Nielsen H, Brunak S, von Heijne G. 1999. Machine learning approaches for the prediction of signal peptides and other protein sorting signals. *Protein Eng* **12**: 3–9.

Niessen CM, Gumbiner BM. 2002. Cadherin-mediated cell sorting not determined by binding or adhesion specificity. *J Cell Biol* **156**: 389–399.

Nollet F, Kools P, van Roy F. 2000. Phylogenetic analysis of the cadherin superfamily allows identification of six major subfamilies besides several solitary members. *J Mol Biol* **299**: 551–572.

Noren NK, Niessen CM, Gumbiner BM, Burridge K. 2001. Cadherin engagement regulates Rho family GTPases. *J Biol Chem* **276**: 33 305–33 308.

Nose A, Nagafuchi A, Takeichi M. 1988. Expressed recombinant cadherins mediate cell sorting in model systems. *Cell* **54**: 993–1001.

Oda H, Tsukita S. 1999. Nonchordate classic cadherins have a structurally and functionally unique domain that is absent from chordate classic cadherins. *Dev Biol* **216**: 406–422.

Oda H, Uemura T, Harada Y, Iwai Y, Takeichi M. 1994. A *Drosophila* homolog of cadherin associated with Armadillo and essential for embryonic cell–cell adhesion. *Dev Biol* **165P**: 716–726.

Oda H, Uemura T, Takeichi M. 1997. Phenotypic analysis of null mutants for DE-cadherin and Armadillo in *Drosophila* ovaries reveals distinct aspects of their functions in cell adhesion and cytoskeletal organization. *Genes Cells* **2**: 29–40.

Pearson WR, Lipman DJ. 1988. Improved tools for biological sequence comparison. *Proc Natl Acad Sci USA* **85**: 2444–2448.

Perl AK, Wilgenbus P, Dahl U, Semb H, Christofori G. 1998. A causal role for E-cadherin in the transition from adenoma to carcinoma. *Nature* **392**: 190–193.

Rawls AS, Guinto JB, Wolff T. 2002. The cadherins fat and dachsous regulate dorsal/ventral signaling in the *Drosophila* eye. *Curr Biol* **12**: 1021–1026.

Redies C. 2000. Cadherins in the central nervous system. *Prog Neurobiol* **61**: 611–648.

Schultz J, Milpetz F, Bork P, Ponting CP. 1998. SMART, a simple modular architecture research tool: identification of signaling domains. *Proc Natl Acad Sci USA* **95**: 5857–5864.

Semb H, Christofori G. 1998. The tumour-suppressor function of E-cadherin. *Am J Hum Genet* **63**: 1588–1593.

Senti KA, Usui T, Boucke K, *et al*. 2003. Flamingo regulates R8 axon–axon and axon–target interactions in the *Drosophila* visual system. *Curr Biol* **13**: 828–832.

Shapiro L, Colman DR. 1999. The diversity of cadherins and implications for a synaptic adhesive code in the CNS. *Neuron* **23**: 427–430.

Sweeney NT, Li W, Gao FB. 2002. Genetic manipulation of single neurons *in vivo* reveals specific roles of Flamingo in neuronal morphogenesis. *Dev Biol* **247**: 76–88.

Takeichi M. 1990. Cadherins: a molecular family important in selective cell–cell adhesion. *Annu Rev Biochem* **59**: 237–252.

Takeichi M, Hatta K, Nose A, Nagafuchi A. 1988. Identification of a gene family of cadherin cell adhesion molecules. *Cell Differ Dev* **25**: (suppl): 91–94.

Tanihara H, Sano K, Heimark RL, St John T, Suzuki S. 1994. Cloning of five human cadherins clarifies characteristic features of cadherin extracellular domain and provides further evidence for two structurally different types of cadherin. *Cell Adhes Commun* **2**: 15–26.

Tepass U, Gruszynski-DeFeo E, Haag TA, Omatyar L, Torok T, Hartenstein V. 1996. shotgun encodes *Drosophila* E-cadherin and is preferentially required during cell rearrangement in the neurectoderm and other morphogenetically active epithelia. *Genes Dev* **10**: 672–685.

Tepass U, Truong K, Godt D, Ikura M, Peifer M. 2000. Cadherins in embryonic and neural morphogenesis. *Nat Rev Mol Cell Biol* **1**: 91–100.

Thompson JD, Gibson TJ, Plewniak F, Jeanmougin F, Higgins DG. 1997. The CLUSTAL_X windows interface: flexible strategies for multiple sequence alignment aided by quality analysis tools. *Nucleic Acids Res* **25**: 4876–4882.

Togashi H, Abe K, Mizoguchi A, *et al*. 2002. Cadherin regulates dendritic spine morphogenesis. *Neuron* **35**: 77–89.

Usui T, Shima Y, Shimada Y, *et al*. 1999. Flamingo, a seven-pass transmembrane cadherin, regulates planar cell polarity under the control of Frizzled. *Cell* **98**: 585–595.

Vogt L, Schrimpf SP, Meskenaite V, *et al*. 2001. Calsyntenin-1, a proteolytically processed postsynaptic membrane protein with a cytoplasmic calcium-binding domain. *Mol Cell Neurosci* **17**: 151–166.

Yagi T, Takeichi M. 2000. Cadherin superfamily genes: functions, genomic organization, and neurologic diversity. *Genes Dev* **14**: 1169–1180.

Zdobnov EM, Apweiler R. 2001. InterProScan — an integration platform for the signature-recognition methods in InterPro. *Bioinformatics* **17**: 847–848.

Zdobnov EM, von Mering C, Letunic I, *et al*. 2002. Comparative genome and proteome analysis of *Anopheles gambiae* and *Drosophila melanogaster*. *Science* **298**: 149–159.

Zhu H, Luo L. 2004. Diverse functions of N-cadherin in dendritic and axonal terminal arborization of olfactory projection neurons. *Neuron* **42**: 63–75.

Copyright © 2005 John Wiley & Sons, Ltd.
